# Traumeel® Epidural Injection: A Viable Alternative to Corticosteroids - A Five-Patient Case Study

**DOI:** 10.7759/cureus.6196

**Published:** 2019-11-19

**Authors:** Dianna Ehlert, Ariel Majjhoo

**Affiliations:** 1 Neurology, Neurointerventional Pain Management, Monroe, USA; 2 Pain Medicine, Neurointerventional Pain Management, Monroe, USA

**Keywords:** traumeel, steroid injections, epidural, low back pain, homeopathy

## Abstract

Epidural steroid injections are the cornerstone of symptomatic relief in select spinal pain conditions and remain the most common interventional procedure; however, many patients with low back pain cannot tolerate corticosteroids due to multiple inherent side effects and contraindications. Plant-based or homeopathic injectable therapies, such as Traumeel®, may be viable alternatives to corticosteroids in providing pain relief for those patients that cannot use corticosteroids in epidural injections.

A chart review case study exploring the effectiveness of Traumeel epidural injections as an alternative treatment to that of epidural corticosteroid injections were performed. We assessed the results of five patients with chronic low back pain who had opted for Traumeel epidural injections for pain relief, report of increasing functional capacity, and the non-expression of side effects. In all cases, pain relief was appreciated at an average of 50% less than chronic pain levels, measured on a numeric rating scale. Additional reports of increased function were consistent throughout all patients in this study. No side effects were reported.

For select spinal pain conditions, Traumeel provided 50% symptomatic pain relief and an increase in reported function via epidural injection without a report of side effects.

## Introduction

The prevalence of low back pain has ranged from 22% to 48% in the adult population and, aside from the common cold, is the primary reason people seek medical attention [1]. Low back pain can be debilitating, causing a progressive decrease in function and a lowered quality of life. The risk of complications and morbidity with untreated acute/chronic low back pain may lead to long-term physical disability and risk of psychological impairment with increasing anxiety, sleep disorders, low morale, loss of control, and loss of autonomy [2].

Although most patients may have nonspecific back pain, several patients are diagnosed with spinal stenosis (secondary to a variety of etiologies) and intervertebral disc bulges/herniations and may present with radicular symptoms from nerve-root irritation and swelling. Inflammation causes impingement on the nerve root and radicular pain can cause discomfort; treatment is needed when this discomfort lowers the quality of life, function, and overall health [2]. When conservative treatments fail, e.g., oral medications and physical therapy, injectable therapies can target drug delivery to the inflamed area (the epidural space).

The epidural space is anteriorly bounded by the posterior longitudinal ligament, posteriorly by the anterior lateral surface of the vertebral lamina and the ligamentum flavum, and laterally bounded by the pedicles of the vertebra and the intravertebral foramen [3]. This space contains neurovascular elements that are important in pain processing. The sensation of pain can further be appreciated when an unregulated inflammatory response is present as this can cause pressure on the nerve. An anti-inflammatory agent is injected into this space, causing a decrease in inflammation and aberrant nerve stimulation, thus relieving pain and ultimately increasing function. 

Epidural steroid injections (ESI) can be considered the cornerstone of treatment in select spinal pain conditions and remain the most common interventional procedure performed by the interventional pain physician. Epidural corticosteroid injections contain drugs that mimic the effects of the hormones produced in the adrenal gland: cortisone and hydrocortisone [4]. Currently, most epidural injections consist of corticosteroids, coupled with an anesthetic, such as Marcaine. These injections are generally well-tolerated and can provide short-term relief for select patients with cervical or lumbar pain [5-6]. However, corticosteroid injections are not without side effects and contraindications stemming from both localized and systemic physiological responses.

Corticosteroid injections have been reported to cause side effects, such as local depigmentation, skin fragility, easy bruising, development of telangiectasia, weight gain, and facial flushing [4-5, 7]. Eventually, the glucocorticoids are absorbed though the epidural venous plexus into systemic circulation where the agents are ultimately metabolized and eliminated from the circulation by the liver and kidneys. Inevitably, every tissue, including skin, neuronal tissue, muscle, and bone, alter their function to some degree when subjected to the glucocorticoid signal [5]. More serious reactions may include osteoporosis and bone changes, hyperglycemia, cardiovascular effects, hypothalamic-pituitary axis suppression, psychiatric effects, infection, and spinal cord infarction [4]. Relative contraindications to corticosteroids include patient preference, previous allergic reaction, recent or upcoming vaccinations, anticoagulant therapy, and chronic conditions, such as diabetes, epilepsy, high blood pressure, or liver, heart, or kidney pathology [8]. Due to the extensive side effect profile, corticosteroid injections are not suited for many patients. 

Homeopathic alternatives, such as the anti-inflammatory compound, Traumeel®, have had a recent resurgence in popularity as they typically do not have the extensive side effect profile found in corticosteroids. An allergy to one or more components of Traumeel has been the only contraindication reported [9]. Traumeel is a fixed combination of diluted plant and mineral extracts that have been available over-the-counter in Europe for over 60 years. In a classic study by Lussignoli et al., it was found that Traumeel decreased systemic interleukin-6 production and reduced edema, off-setting an unregulated inflammatory response [10]. In an in vitro study on human T-cells, monocytes, and gastrointestinal epithelial cells, Traumeel was found to inhibit secretion of the pro-inflammatory mediators interleukin-1 beta (IL-1β), tumor necrosis factor-alpha (TNFα), and interleukin-8 (IL-8) [8]. This suggests that Traumeel may have the capability of stabilizing immune cells. Although the ingredients of Traumeel have been used for many years for therapeutic purposes (e.g., Echinacea angustifolia and Echinacea purpurea to increase mesenchymal defenses or “boost the immune system” [8]), the effect of Traumeel was found to be greater than the “sum” of the active components, with multiple components acting on various phases of the inflammation response, suggesting a synergistic interaction [9-10].

Traumeel has been indicated to treat various inflammatory conditions with comparable efficacy to nonsteroidal anti-inflammatory drugs (NSAIDs) via oral, topical, and injectable preparations [8-9]. There is no known current research regarding the efficacy of Traumeel in epidural injections for short-term relief of back pain or studies comparing Traumeel to corticosteroid injections, although the Traumeel in Rotator Cuff Syndrome (TRARO) study protocol outlined a potential method for comparison in patients with rotator cuff syndrome [11].

Herein, we describe five patients seeking back pain relief who had opted for Traumeel injections due to a contraindication to or a preference against steroids. It is hoped that this will lay the groundwork for further studies into the efficacy of Traumeel in epidural injections to allow for additional pain-relieving options, especially in patients unable to tolerate corticosteroid injections.

## Case presentation

The procedure was performed in an outpatient setting. The option of conscious sedation was extended as some patients can experience extreme anxiety related to procedures containing needles. 

Preop instructions included: hold all non-steroidal anti-inflammatories (NSAIDs) for at least two to four days and other blood thinners (with prescribing physician consent) for at least one to seven days, do not eat or drink anything for two hours before the procedure for local anesthesia only, and do not eat or drink anything for eight hours before a conscious sedation procedure. A driver must be present for conscious sedation procedures and may be recommended for other non-sedation cases. Chronic medications, aside from blood thinners and NSAIDs, can be taken the morning of the procedure. Informed consent was obtained with benefits, risks, and alternatives to the procedure explained. 

The epidural injection (EI) procedure

The patients were placed in the prone position. The overlying skin was widely prepped with chlorhexidine gluconate and isopropyl alcohol (Chloraprep One-Step), allowed to dry, and then a sterile drape was placed. A local anesthetic of 5 ml of 2% lidocaine via a 25-gauge needle was administered between the intended epidural space. Under fluoroscopy, a 20-gauge Tuohy Weiss needle (Halyard Alpharetta, GA) was used to enter the intended epidural space using a loss of resistance technique. Multiple fluoroscopic views confirmed the proper positioning of the needle tip before injecting non-ionic contrast dye for additional confirmation of correct placement. Aspiration was confirmed to be negative for cerebral spinal fluid or blood with no occurrence of paresthesia before depositing a solution of 2.2 ml of Traumeel (Table 1), 0.5 - 1 ml of 0.25% Marcaine, and 2 ml of 0.9% normal saline solution.

**Table 1:** Traumeel Components

Traumeel Active Extract Ingredients:	Amount per Ampoule for injection (per 2.2 mL)
Achillea millefolium (milfoil)	2.2 μL
Aconitum napellus (monkshood)	1.32 μL
Arnica montana (mountain arnica)	2.2 μL
Atropa belladonna (deadly nightshade)	2.2 μL
Bellis perennis (daisy)	1.1 μL
Calendula officinalis (calendula)	2.2 μL
Matricaria recutita (chamomile)	2.2 μL
Echinacea angustifolia (narrow-leaved coneflower)	0.55 μL
Echinacea purpurea (purple coneflower)	0.55 μL
Hamamelis virginiana (witch hazel)	0.22 μL
Hepar sulphuris calcareum (burnt flower of sulfur/inner layer of oyster shells or calcium carbonica)	2.2 μL
Hypericum perforatum (St. John's Wort)	0.66 μL
Mercurius solubilis	1.1 μL
Symphytum officinale (comfrey)	2.2 μL
Inactive Ingredients:	
Water for injection	2,179.10 μL
Sodium chloride	19.40 μL

The patients were brought to the recovery room and observed for complications. Subsequently, they were discharged with a return office visit scheduled for evaluation. The evaluation consisted of a subjective assessment of pain relief on the numeric rating scale (NRS) of zero to 10 with zero as no pain and 10 as the worst pain imaginable, functionality changes, and the appearance of side-effects. 

Case studies

Patient 1

A 51-year-old right-handed male presented with a history of diabetes mellitus (DM), migraine headaches, and cervical and lumbar pain. He was referred by neurosurgery for cervical medial branch blocks (CMBBs) at C2-C4 for suspected cervicogenic headaches and epidural steroid injections (ESI) at L4-L5 for a bulging disc and low back pain. The headaches were associated with nausea, vomiting, and photophobia with additional cervical radiculopathy symptoms. As his blood sugars had dangerously increased to the range of 500+ from corticosteroid injections in the past, the patient opted for Traumeel injections as a viable alternative to corticosteroid injections. The CMBB Traumeel injection proved to be a therapeutic injection as he responded with a significant pain reduction and a decrease in the frequency of the headaches. He then underwent a Traumeel epidural injection series of two injections, two weeks apart, which reduced his low back pain to tolerable levels. The original pain did return two months later, and he eventually was referred back to his neurosurgeon.

Patient 2

A 33-year-old male presented with upper thoracic non-radicular pain after a motor vehicle accident (MVA) 12 months prior. He complained of constant throbbing pain with an intermittent sharp quality, rated at 9/10 on the NRS. After no improvement with physical therapy, we discussed an epidural injection at T2-T3 where imaging showed a 4 mm disc bulge which was compressing the thecal sac. The patient and his wife practiced the principles of homeopathy and favored natural products. Corticosteroid injections were refused in favor of Traumeel (the homeopathic alternative). Although high anxiety related to injection therapy and an initial vasovagal reaction occurred, he successfully completed a two-injection series with a pain reduction of > 50% (NRS reported as 4/10 at its highest) and increased exercise tolerance for greater than six months.

Patient 3

A 52-year-old female presented with lower right-sided back pain and right radicular symptoms, extending to the mid-calf. She described it as a sharp pain with shooting pains four to five times per day, reporting an NRS rating of 7/10. Magnetic resonance imaging (MRI) revealed a disc herniation at L4-L5 with impingement on the exiting nerve root. She responded well to transforaminal corticosteroid epidural injections, reporting an NRS rating of 4-5/10, a 50% increase in function, and a reduction of pain medication after a two-injection series. Although she appreciated the three-to-four-month relief she received, she stated that the corticosteroids were causing weight gain, and this was "not a fair trade.” To mitigate the side effect of weight gain commonly experienced with corticosteroids, she opted to change her injectate to a Traumeel and Marcaine solution. She reported similar pain relief with an NRS rating of 5/10 and retained a 50% increase in function with no change in pain medications and without the side effect of weight gain.

Patient 4

A 71-year-old female presented with a history of chronic lower lumbar spinal pain and right radicular symptoms. She was diagnosed with post-laminectomy syndrome after undergoing multiple lumbar surgeries without full alleviation of chronic pain. We discussed caudal epidural injections of corticosteroids. She professed a reaction to corticosteroids in the past causing palpitations and headaches; therefore, corticosteroids were not recommended. She opted for an injection using Traumeel, Marcaine, and normal saline. She reported three days of greater than 50% pain relief with increased function and pain tolerance; she denied any side effects to the Traumeel injections.

Patient 5

A 45-year-old female with a history of bipolar disorder presented with new-onset of thoracic pain. Disc bulges were seen on MRI, with the most prominent site at T11-12. She stated she was unable to tolerate corticosteroids due to exacerbations in her mental illness. She opted for an injection series with Traumeel; no change in her mental status was observed directly after the injection or subsequently reported. In conjunction with physical therapy, she reported 40% - 50% relief of her pain, increased range of motion and functionality, and was overall satisfied. No additional treatments, such as medications or surgical referrals, were needed.

## Discussion

Back pain can be debilitating, causing a progressive decrease in function and a lowered quality of life. ESIs have been a common treatment for a variety of spinal conditions, but corticosteroids have a myriad of inherent side effects. An alternative medication is needed, especially for patients with significant contraindication to corticosteroid injections, to provide pain relief via epidural injection (EI) with a low occurrence of side effects.

We performed a chart review on five patients that opted for Traumeel epidural injections over steroid injections. We focused on the expression of pain relief, report of increasing functional capacity, and the non-expression of side effects. In all cases, significant pain relief was appreciated after an EI with Traumeel (at an average of 50% less on the NRS than chronic pain levels). This is comparable to the classic placebo-controlled double-blind trial in which patients received intra-articular injections of either Traumeel or a placebo to determine the reduction in pain; the Traumeel group had a 25.3% higher occurrence of no pain reports after eight days [8]. Reports of increased function were consistent throughout all patients in this study, which was in line with classic and recent studies that have shown that Traumeel increases joint mobility, translating to increased ease of movement [8-9, 12]. No Traumeel-related side effects were seen. This is congruent with multiple studies of Traumeel therapies via oral, topical, and injectable administration [8-9, 12]. A vasovagal reaction occurred in one patient, but this was thought to be due to pain and anxiety from the procedure. With his second injection, light sedation was administered, and the procedure was tolerated well with no vasovagal reaction or other side effects reported.

The limitations of this study are not uncommon to case reviews in general; a small study size containing patients of varying age and etiology with narrow background information in a non-randomized setting without a placebo control group. The statistical analysis was inherently skewed by a small number of participants and a prevalent issue in pain management (pain and level of functioning are inherently subjective and not reported consistently). A standardized pain and functioning scale would be helpful in future studies. As the need for an alternative medication stems from the inability of the patient to tolerate corticosteroids, it was, therefore, impossible to compare Traumeel with corticosteroids in all cases. However, Patient 3 related similar pain relief and functional increase qualities with Traumeel versus corticosteroids. The TRARO study was proposed in 2015 to assess the efficacy and safety of Traumeel versus dexamethasone versus placebo; no results of this proposed study have been published [11].

There was a noticeable lapse of research published on Traumeel from 2007 to 2011 with sporadic publications after 2011. There is no known research assessing Traumeel versus corticosteroids in epidural administration. A double-blind randomized control trial as to the efficacy of Traumeel versus corticosteroids is warranted. Research into the efficacy of Traumeel for medial branch block injections may also be warranted as Patient 1 experienced pain relief with a CMBB injection as well as the EI.

## Conclusions

Although side effects from epidural corticosteroid injections are usually not lethal, they can be very unsettling to patients and may carry significant morbidity. Traumeel is an effective and well-tolerated alternative to corticosteroids in providing pain relief and increased function via epidural injection with a low occurrence of side effects. Additional studies are needed to determine the full benefits of Traumeel-based injection therapies as the risk-benefit scale tips toward efficacious plant-based medications.
